# Immune parameters monitored during the production period of laying hens managed with or without single-dose vaccination against erysipelas

**DOI:** 10.1186/s12917-026-05512-w

**Published:** 2026-05-01

**Authors:** Eva Wattrang, Katarina Näslund, Lena-Mari Tamminen, Robert Söderlund, Tina Sørensen Dalgaard, Désirée S. Jansson, Helena Eriksson

**Affiliations:** 1 Department of Microbiology, Swedish Veterinary Agency, Uppsala, Sweden; 2 Department of Clinical Sciences, Swedish University of Agricultural Sciences, Uppsala, Sweden; 3 Department of Animal Science, Aarhus University, Tjele, Denmark; 4 Department of Animal Health and Antimicrobial Strategies, Swedish Veterinary Agency, Uppsala, Sweden

**Keywords:** Erysipelas, *Erysipelothrix rhusiopathiae*, Laying hen, IgY, Leukocyte counts, Mannose binding lectin

## Abstract

**Background:**

Erysipelas, caused by infection with *Erysipelothrix rhusiopathiae* (ER), is an emerging disease in non-cage housed laying hens. According to Swedish field experience outbreaks seem more common in older flocks and may also occur in flocks vaccinated against erysipelas. This cross-sectional study aimed to assess single-dose vaccination outcome in laying hens with respect to antibody titres to ER and to monitor selected immune parameters to identify putative risk factors for erysipelas. Blood samples were collected from laying hen flocks at 30, 50 and 75 weeks of age. At each time point, 5 unvaccinated flocks and 5 flocks that had been vaccinated against erysipelas once at placement were sampled (20 hens/flock, total n=600).

**Results:**

The results showed that the majority of hens were positive for IgY to ER and that the antibody titres were higher in older hens irrespective of vaccination status. Vaccinated hens had significantly higher IgY titres to ER compared to those of unvaccinated hens. This difference was most prominent for the youngest age category. Among the different leukocyte populations studied, blood heterophil, monocyte and γ/δTCR+ T-cell counts were significantly higher in younger hens. Also, serum MBL-levels were significantly higher in younger hens and MBL-levels were positively correlated to heterophil and monocyte counts at the individual level.

**Conclusions:**

Taken together, results indicate that ER or antigenically similar bacteria are common in the hen environment and that this exposure results in antibodies recognising ER that are present at higher levels in older laying hens. In addition, a higher general pathogen load/level of subclinical infections was indicated by altered leukogram patterns and increased MBL-levels in the youngest age category. Nonetheless, among the parameters studied we found no evidence to suggest that antibody responses to single-dose vaccination should fail or why older flocks seem more susceptible to erysipelas outbreaks.

**Supplementary Information:**

The online version contains supplementary material available at 10.1186/s12917-026-05512-w.

## Background

Erysipelas, caused by infection with the bacterium *Erysipelothrix rhusiopathiae* (ER), presents as septicaemia and sudden death in laying hens and may often cause outbreaks with high mortality in affected flocks (reviewed in [1]). In modern egg production erysipelas outbreaks are perceived as emerging in e.g., several European countries [1]. The emergence of this problem is associated with changes in laying hen housing systems from battery cages to barn production and an increased risk of erysipelas outbreaks has been observed for flocks with outdoor access [1]. A recent analysis of erysipelas outbreaks in Sweden between 1998 and 2023 also showed that the mean flock age at outbreaks was > 60 weeks and approximately 30% of outbreaks occurred in flocks vaccinated against the disease [2]. Nevertheless, important knowledge on this disease in laying hens e.g. on the source of infection, on immune responses against infection and on effective prophylactic measures is still missing.

The ER bacterium is a Gram-positive facultative anaerobic rod and it may infect a wide range of hosts including many mammalian and avian species with or without causing clinical disease (reviewed in [1, 3]). The source of infection in a particular case is often unidentified although it is known that the bacterium may survive in the environment for months and at least for pigs it is thought that healthy carriers is an important source of infection [3–5]. For chickens there are likewise reports of ER in healthy birds, e.g. one early report on healthy ER carriers [6] and two reports on detection of ER at slaughter of healthy birds [7, 8]. Preliminary results also show the presence of ER in the barn environment of some healthy Swedish laying hen flocks [9]. Moreover, serological evidence indicates that exposure to ER may be common in healthy chickens [10–13].

Among domestic animals, acute disease caused by ER infection is most well described for pigs and turkeys and in these species vaccination has also long been used to control the disease (reviewed in [14]). Vaccination against erysipelas is generally perceived as effective at least to control acute disease while chronic manifestations of the disease seem more difficult to prevent. Moreover, evidence of vaccine failure in e.g., pigs have been reported and indications that this may be due to mismatches between vaccine and outbreak ER serovars have been put forward (reviewed in [14]). At least in mammals it is commonly thought that induction of antibodies to ER is the major protective mechanism in vaccine responses (reviewed in [14, 15]). However, conclusive evidence that antibodies confer vaccine-induced or naturally acquired protection against erysipelas in chickens have to our knowledge not been put forward (reviewed in [1]). In fact, due to the intracellular phases of ER infection it is highly likely that a Th1-type response including e.g. interferon-γ induced activation of phagocytic cells, also plays an important part of protective acquired immune responses against this disease [1].

For laying hens there is no registered vaccine against erysipelas but vaccines approved for other species are being used off-label, e.g. an inactivated vaccine registered for use in turkeys is used in Denmark (S. Kabell, personal communication) and in Sweden [16]. The manufacturer’s instructions for this vaccine are that turkeys should be vaccinated twice four weeks apart for protection with onset of immunity from six weeks after the second vaccination and lasting 23 weeks after the second vaccination (Nobilis Erysipelas, MSD Animal Health; https://www.msd-animal-health-hub.co.uk/Products/Nobilis-Erysipelas). In Sweden, the routines for vaccination of laying hens against erysipelas with this vaccine are either that new flocks on premises where clinical outbreaks of erysipelas previously have occurred are vaccinated once at placement at the premises (at approx. 14–16 weeks of age) or, less commonly, that a flock is vaccinated once during an outbreak of clinical erysipelas [2]. Vaccination of subsequent flocks on farms that have experienced erysipelas outbreaks is generally perceived as successful, although as mentioned, outbreaks in vaccinated flocks do occur. The outcome, with respect to antibody production, of this vaccination routine has never been evaluated and one hypothesis for outbreaks in vaccinated flocks that has been put forward was that antibody responses could be insufficient or decreasing with time due to the single-dose vaccination regime. To begin addressing this concern, one of the aims of the present study was to assess if it was possible to detect vaccine-induced antibody responses to ER in Swedish laying hen flocks at different time-points during the production period. Even if antibodies to ER may not be a correlate to vaccine induced protection per se, they are a sign that the immune system has responded to the vaccination. Because high throughput methodology to study ER-specific T-cell responses is still lacking we found it justifiable to use antibody responses as a correlate of vaccine exposure in the current study.

In addition, we also monitored a selection of immune parameters in these hens with the objective to screen for potential clues, such as signs of general immune suppression, increased subclinical infections/pathogen load or decreased opsonic capacity, that could explain why erysipelas outbreaks occur at higher frequency late in the production period. For this purpose, we monitored differential leukocyte counts including extended phenotyping of some lymphocyte subpopulations. Different patterns of circulating leukocytes are commonly used to assess the general status of an individual’s immune system where alterations can give indication of e.g. infections [17] or stress responses [18]. We also measured serum levels of the acute phase protein mannose binding lectin (MBL), a C-type collectin and soluble pattern-recognition receptor of the innate immune system [19, 20]. Increased levels of MBL in the circulation are an indicator of ongoing infections [21–25]. Finally, we performed an in vitro ER leukocyte adhesion assay where bacterial adhesion, i.e. the first step of phagocytosis, to different leukocyte populations was quantified in whole blood cultures. Immune cell phagocytic clearance of infectious agents is an important effector mechanism in bacterial infections [17]. In the current assay, the amount of bacteria that adheres to the immune cells depends on both bacterial, e.g. capsular properties, and host factors, e.g. level of opsonisation. Because it is performed in whole blood cultures results can reflect the presence of opsonins such as complement factors and antigen specific antibodies in the circulation and thereby give an estimate of the individuals opsonic capacity. With this method we were for example able to detect increased adherence of *Escherichia coli* in cultures from experimentally *E. coli* infected chickens, which was likely due to antibody opsonisation because it coincided in time with the appearance of infection induced *E. coli*-specific antibodies in the circulation [26].

## Materials and methods

### Flocks and experimental design

The study had a cross-sectional design and comprised 29 commercial flocks of Dekalb White Leghorn-type laying hens. Flocks were sampled at either of three time points during the production period; early (approx. 30 weeks of age), middle (approx. 50 weeks of age) and late (approx. 75 weeks of age). Twenty-eight flocks were sampled on one occasion only and one flock was sampled twice (at 30 and 50 weeks). In each age category, five flocks vaccinated against erysipelas and five flocks not vaccinated against this disease were sampled. Thus, the experiment comprised samples from six groups of five flocks as described in Table 1. Vaccination was performed once at placement at approx. 14–16 weeks of age with the inactivated vaccine Nobilis Erysipelas (MSD Animal Health). All flocks were housed with outdoor access either in organic production, *n* = 25, or free-range with outdoor access, *n* = 4 (1 flock in each group; 30 weeks unvaccinated, 30 weeks vaccinated, 50 weeks unvaccinated and 50 weeks vaccinated, respectively). Flocks were sampled between April and October 2019 and all flocks were reported as healthy by farmers at the time of sampling. For reference, in 2019 19% of the whole Swedish laying hen population were housed with outdoor access (16% in organic production and 3% free-range with outdoor access; personal communication, Lannhard Öberg, Swedish Board of Agriculture, 2025). Inclusion criteria for the selection of participating flocks were: farmer’s willingness to participate, flock age at sampling (desired age ± 5 weeks), known erysipelas vaccination status, Dekalb White hybrid, outdoor access, a clinically healthy flock and farm location i.e. within a distance of the laboratory of four hours driving by car. It cannot be excluded that selection bias may have been introduced by these criteria. At the time of sampling, flock data on e.g. production parameters were collected (Table 1 and Additional file 1).

**Table 1:** Flock group mean values (mean ±1SD and range) for selected numerical variables of the flocks included in the study

Flock group	Age^a^ (weeks)	Facility in use^b^ (years)	Outdoor time^c^ (weeks)	Mortality^d^ (%)	Laying rate^e^ (%)	Feed^e^ (g/chicken/day)	Water^e^ (ml/chicken/day)
30 weeks unvaccinated^f^	29.4 ± 1.8 27–31	12.2 ± 4.6 9–19	6.6 ± 1.1 5–8	1.2 ± 1.3 0.2-3.0	95.6 ± 1.1 94.0-96.7	115.4 ± 7.6 106–125	194.0 ± 24.1 170–225
30 weeks vaccinated^f^	30.0 ± 1.9 28–32	10.0 ± 6.8 4–21	6.8 ± 3.0 2–10	1.1 ± 0.7 0.1-2.0	91.9 ± 6.4 83.0-97.7	117.4 ± 5.7 110–123	194.8 ± 14.6 175–210
50 weeks unvaccinated	50.4 ± 3.2 46–54	15.6 ± 11.4 4–34	15.0 ± 10.4 1–24	1.9 ± 0.5 1.1–2.4	94.4 ± 1.5 92.0–96.0	118.8 ± 4.6 112–122	204.5 ± 18.3 190–228
50 weeks vaccinated	51.6 ± 1.8 50–54	11.4 ± 6.3 4–21	9.6 ± 7.6 2–19	2.1 ± 0.6 1.2–2.7	95.9 ± 0.6 95.0-96.5	120.0 ± 2.1 117–122	215.4 ± 15.7 195–234
75 weeks unvaccinated	76.0 ± 0.7 75–77	13.0 ± 6.8 5–21	14.6 ± 7.8 3–23	5.4 ± 3.0 2.6-9.0	83.3 ± 3.5 80.0–89.0	115.6 ± 5.8 110–125	181.3 ± 8.5 170–190
75 weeks vaccinated	74.4 ± 1.5 72–76	16.2 ± 1.9 13–18	15.6 ± 8.8 3–26	4.5 ± 1.5 2.3-6.0	85.4 ± 4.3 79.7–90.0	118.8 ± 2.2 117–122	182.2 ± 6.2 177–189

a – Age of flock at sampling

b – Number of years the chicken house had been in use at the time of sampling

c – Number of weeks the flock had had access to the outdoor range (due to weather conditions) during the 2019 season at the time of sampling

d – Total cumulative mortality recorded for the flock from placement until the time of sampling

e - Egg production and feed and water consumption recorded at the time of sampling

f - Unvaccinated/vaccinated against erysipelas at the time of placement for details se Materials and Methods

### Sample collection

Blood samples from 20 hens in each flock were collected from the jugular vein and blood from each hen was distributed into a sterile blood collection tube with 34 I.U. lithium heparin as additive (#368494, BD Vacutainer ^®^, BD Life Sciences) and into a sterile test tube without additives. Individual hens were selected at random and inclusion criteria were normal body condition and a clinically healthy status. Chilled blood samples were transported to the laboratory and the immunolabelling and ER adhesion assays were performed within 8 h of blood sampling. Sera were stored at -20 °C until analysis which was performed during November 2019.

### ELISA for detection of antibodies to ER

An in-house ELISA for detection of antibodies to ER in chicken serum was set up based on an earlier described protocol [27] and previously described in detail [10, 25]. Data from specific pathogen free (SPF)-reared chickens in the latter studies [10, 25] were used to calculate sensitivity and specificity for the ELISA. For this calculation 14 uninfected SPF-chickens sampled at 41–45 days old were classified as true negatives and a cut-off titre value of 81 (group mean value + 2SD) for positive titres were calculated from data from these chickens. Ninety-six experimentally ER infected SPF-chickens sampled at 10, 11 or 15 days after infection (33–45 days old) were classified as true positives. With the titre results from these two groups a sensitivity of 96.0% (90.1%-98.9%; 95% confidence interval) and a specificity of 93.3% (68.1%-99.8%) were calculated using MedCalc Software Ltd. Diagnostic test evaluation (https://www.medcalc.org/en/calc/diagnostic_test.php; Version 23.5.1; accessed March 31, 2026).

For the present study, the ELISA coating antigen was prepared from ER strain 13-ALD025893, which belongs to clade 2 and in silico serotype 5 [2]. All serum samples were titrated in 2-fold steps starting at dilutions 1:100 or 1:1000 depending on antibody concentration, to achieve a dilution curve. For each sample the A_450_ values were plotted against the sample dilution and the equation for the linear part of the curve was determined by regression analysis. Antibody titres were calculated as the dilution that would achieve an A_450_ value of 1. The inter-assay coefficient of variability for the positive control in the current study was 12%, *n* = 44.

### Blood leukocyte counts

Absolute counts of heterophilic granulocytes, monocytes, lymphocytes and thrombocytes (Panel 1: Additional files 2 and 3) and lymphocyte subpopulations; TCRg/δ+ cells, TCRg/δ-CD8αβ+, cells, i.e. cytotoxic T-cells (CTL), and TCRg/δ-CD8αα+ cells (Panel 2: Additional files 2 and 4), in heparinised whole blood samples were determined using a no-lyse, no-wash flow cytometry based method previously described [25]. In brief, 25 µl of heparinised blood was diluted in FACS-buffer, i.e. phosphate buffered saline (PBS) supplemented with 0.2% bovine serum albumin (BSA; Sigma-Aldrich), 0.2% sodium azide, 0.05% normal horse serum (Sigma-Aldrich) and 2 mM EDTA and immunolabelling was performed using two panels of fluorochrome conjugated antibodies (Additional file 2). The gating strategies to define different leukocyte populations are shown in Additional files 3 and 4. The number of events counted in the bead gate (123count eBeads, #01-1234-42, Invitrogen, Thermo Scientific) was according to the manufacturer's recommendations at least 1000 and was used to determine the volume of blood sample analysed and calculate absolute numbers of the leukocyte populations. Flow cytometry was performed using a BD FACSVerse™ (BD Biosciences), equipped with 488 nm blue, 633 nm red and 405 nm violet lasers and results were analysed using the FACSDiva (BD Biosciences) software. Single-stained compensation controls and fluorescence minus one (FMO) negative controls were included in the assays. Titrations of all antibodies were performed to determine optimal labelling conditions prior to the experiment.

### ER leukocyte adhesion assay

Adhesion of fluorescently labelled ER to chicken leukocytes was assessed with a method based on a previously described flow cytometry assay for phagocytosis in chicken whole blood samples [28]. For fluorescence labelling of ER the CellTrace™ Far Red Cell Proliferation kit (#C34572, Invitrogen, Thermo Fischer Scientific) was used as earlier described for labelling of live Gram-positive bacterium *Orientia tsutsugamushi* [29]. Titration of the dye was performed before the experiment to determine the optimal Far Red concentration for ER labelling.

For labelling of ER, strain 13-ALD025893 was maintained in culture on horse blood agar (#B341180; Swedish Veterinary Agency, Uppsala, Sweden). Prior to labelling, bacteria from an 24 h culture on horse blood agar were cultured for 24 h at 37 °C on a shaker in tryptic soy broth (#B321730, Swedish Veterinary Agency) supplemented with 0.1% Tween 80, 0.1% D-glucose and 20 mg/L L-tryptophan. Bacteria were pelleted at 3220 x *g* for 10 min, the supernatant was removed and the bacteria were resuspended in 300 µl 5 mM working dilution of Far Red dye per an estimated 10^8^ cfu ER. Immediately prior to labelling the Far Red dye was diluted into a 5 mM working dilution in 37 °C tempered PBS according to the manufacturer’s instructions. The suspension was incubated at 37 °C on a shaker in the dark for 20 min. Subsequently five-times the staining volume of RPMI medium (RPMI 1640, Swedish Veterinary Agency) supplemented 5% foetal calf serum (Gibco^®^ #10082147, ThermoFisher Scientific) was added and the suspension incubated at room temperature for 5 min. Bacteria were then pelleted at 3220 x *g* for 10 min, the supernatant was removed and the bacteria were resuspended in RPMI without additives and stored at 4 °C in the dark until use. Numbers of labelled ER were determined by a 10-fold serial dilution of labelled bacteria; 100 µl volumes of each dilution were spread on agar plates, cultured for 48 h at 37 °C, ER colonies were counted and cfu per ml was calculated.

The ER adhesion assay was performed in round-bottomed anti-cell adhesion 96-well cell culture plates (#83.3925.500, TC plates susp R, SARSTEDT). To each well 40 µl RPMI supplemented with 2mM L-glutamine, 10 µl heparinised blood, 50 µl panel 1 of fluorochrome conjugated antibodies (Additional file 2) diluted in RPMI with L-glutamine and 25 µl of Far Red labelled ER diluted to 10^8^ cfu/ml in RPMI with L-glutamine. Plates were incubated at 40 °C for 15 min where after 15 µl of 20 mM EDTA in PBS was added to each well (gives a final concentration of 2 mM EDTA) and the plates were incubated for 10 min at room temperature. Then 30 µl of 4% PFA in PBS was added to each well, cells were suspended by pipetting up and down and the content of the wells was transferred to FACS tubes containing 300 µl FACS buffer. Samples were stored at 4 °C until analysis by flow cytometry as described above. Flow cytometry data were recorded at reduced flow rate until 1000 events in the monocyte gate were collected and gating strategies to define different leukocyte populations and proportions of adhered ER are shown in Additional file 5.

### ELISA for detection of chicken MBL

The MBL serum concentration was measured using an earlier described in house ELISA based on the anti-chicken cMBL antibody HYB182-01 from BioPorto A/S [21, 30].

### Data analysis

Statistical analysis of data was performed in R (version 4.3.0) [31]. To explore the impact of flock level variables on antibodies, linear mixed effects models were fitted using the *lme4* package [32]. Antibody titres were log-transformed to improve residual normality. Vaccination status and age were considered variables of primary interest and included as fixed effects and flock as a random effect to account for clustering. Age groups were categorised as early (30 weeks), middle (50 weeks) and late (75 weeks). Additional flock level variables (Additional file 1) were considered as potential fixed effects in the model. Each flock level variable was assessed by a combination of likelihood ratio test and Akaike Information Criterion (AIC). Variables were retained in the model if they improved model fit (ΔAIC ≤ − 2) or showed evidence of association (*p* < 0.05). Residual plots were used to evaluate normality and homoscedasticity and interaction between age group and vaccination explored to detect effect modification. Multicollinearity was evaluated using variance inflation factor (values > 5 considered problematic) [33].

After defining the multivariable mixed-effects model, variables that had been excluded during the model-building process were reintroduced individually to the final model to evaluate their potential role as confounders. If introduction of a variable resulted in a change of > 10% of the estimated effects of model estimates the variable was considered a potential confounder. Such variables that could plausibly be interpreted as possible confounders (e.g. proxies of management practices or infection pressure) were retained in the model, provided they did not compromise model stability. Predicted values visualising the effect of vaccination and age group on antibody titres from the final mixed-effects model were generated to aid interpretation using the packages *ggeffects* and *ggplot2* [34, 35].

An exploratory investigation of associations between levels of antibodies, vaccination and immune parameters across the age groups was performed using cluster analysis (clumix) [36, 37]. Observations were clustered by similarity (based on Gower’s general similarity coefficient) and variables clustered using association measures (absolute Spearman correlation coefficient, absolute Goodman and Kruskal’s gamma coefficient). In addition, univariable associations between vaccination and immune parameters as well as age and immune parameters were calculated using the non-parametric Kruskall-Wallis rank sum test (table one) [38].

Correlation of immunological parameters on the individual hen level was illustrated using Pearson coefficients.

## Results

### Flock parameter overview

Data on production parameters, management practices and health were collected from flocks in the study (Table 1 and Additional file 1). Flock cumulative mortality was positively correlated to age and negatively correlated to egg production. Mortality was slightly higher in the 50-week age category compared to the 30-week-old flocks and 2-fold higher in week 75 compared to the younger age groups (Table 1). Similarly, the laying rate was lower in 75-week-old flocks. In general, the 30-week-old flocks had had shorter use of the outdoor run at the time of sampling compared to the older flocks and more of the 50- and 75-week-old flocks were sampled at the end of the sampling period and therefore had had use of the outdoor runs for longer at the time of sampling. Taken together, flocks included in the study showed production and health parameters within expectation for their respective age groups and their profiles were reasonably distributed between the unvaccinated and vaccinated groups (Table 1).

### Antibodies to ER in vaccinated and unvaccinated hens

**Fig. 1:**
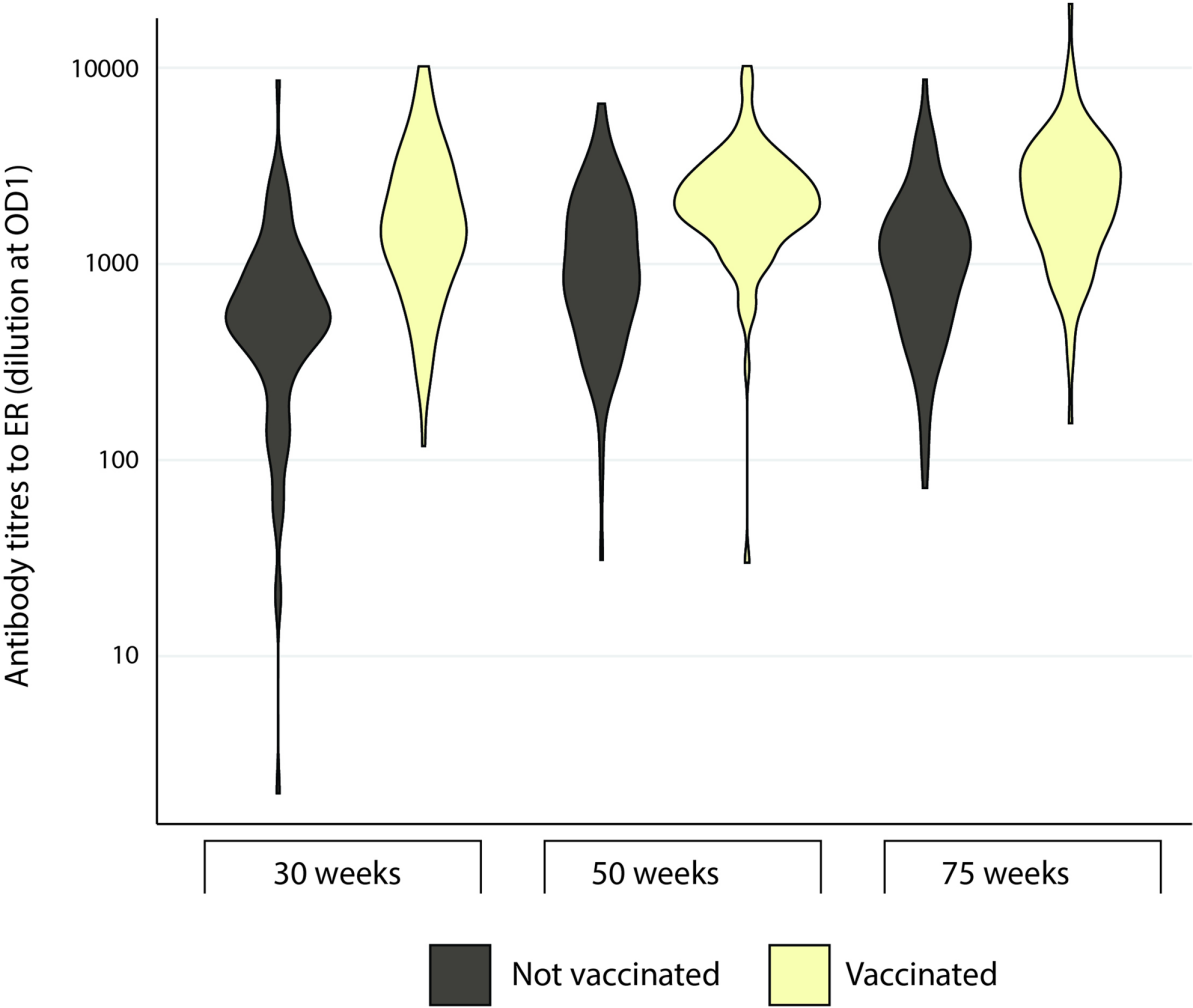
Serum IgY titers to ER. Violin plots showing the maximum, minimum and frequency of titres to ER in serum from laying hens vaccinated against erysipelas (light yellow) and unvaccinated laying hens (grey) in the indicated age categories. Data is shown as group plots with 94 ≥ n ≤ 100 for each group, missing values, i.e.<100, were due to insufficient amount of serum

Levels of IgY to ER were quantified using ELISA methodology. The results showed that all hens in the study were positive for IgY to ER regardless of whether they had been vaccinated against erysipelas or not (Fig. 1). The IgY titres to ER were significantly higher in vaccinated compared to unvaccinated hens as well as higher in older (week 50 and 75) compared to younger hens regardless of vaccination (Table 2; Fig. 1). Flock explained 0.02 of the variance compared to 0.74 residual variance, indicating large variability within flocks. Individuals with very high IgY titres (> 5 000) were observed in almost all flocks regardless of age and vaccination status (Additional file 6).

**Table 2:** Associations between flock-level characteristics and antibody levels to ER estimated using a linear mixed-effects regression model. Antibody levels from 594 (missing values were due to insufficient amount of serum) hens sampled in 30 flocks were analysed on the log(1 + outcome) scale. Flock was included as a random intercept to account for clustering. Estimates represent adjusted fixed effects with 95% confidence intervals

Variable		*n*	Estimate	Confidence interval	*p*-value*
Age	30 weeks	200			< 0.001
	50 weeks	196	0.49	0.26–0.71	
	75 weeks	198	0.2	-0.17-0.57	
Vaccination ER	No	294			< 0.001
	Yes	300	0.80	0.59-1.00	
Vitamin supplement	No	317			0.13
	Yes	277	0.15	-0.05 -0.35	
Total egg production	*continuous*	91.2 *(85.3–96)*	-0.03	-0.06–0.003	0.07
Treatment against *Dermanyssus gallinae*	No	158			0.09
	Yes	436	-0.2	-0.45–0.04	

*Wald type 3

### Other flock parameters and antibodies to ER

In addition to age and vaccination status, two flock level variables (vitamin supplementation and laying rate) were retained in the final model as they improved model fit (assessed by AIC). However, confidence intervals were broad and included null (Table 2).

Results from the linear mixed model were robust to sensitivity analyses excluding treatment against *Dermanyssus gallinae*. The inclusion of this variable altered the estimated vaccination effect and because it could be considered a possible confounder, for example a proxy for general preventive management and/or infection pressure, it was retained in the final model. It should be noted that the effect estimate for vaccination was larger in the model that did not adjust for this variable (0.88 compared to 0.80). Residual diagnostics indicated acceptable model fit, with no evidence of overdispersion or influential outliers affecting inference.

### Immune parameters, age and antibodies/vaccination to ER

**Fig. 2:**
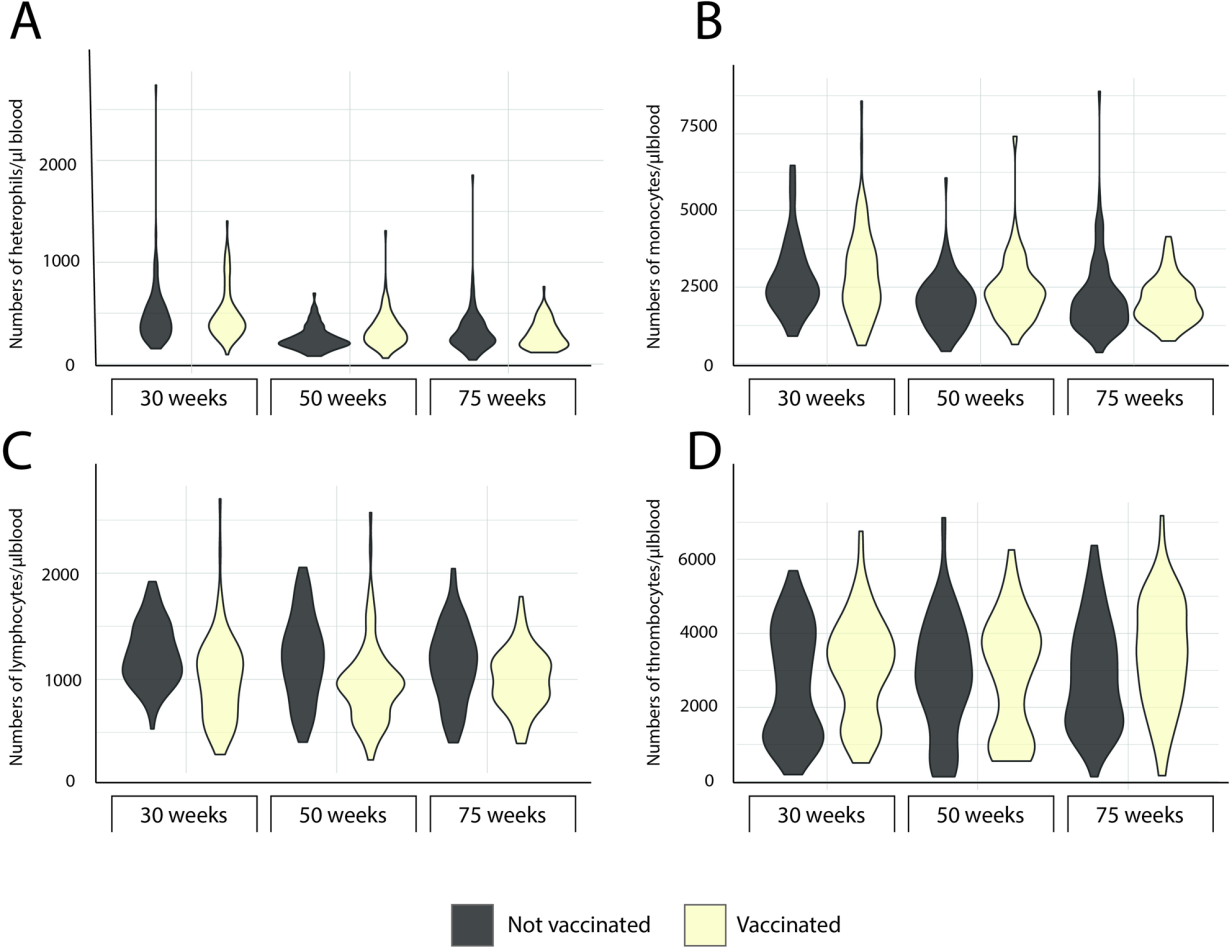
Blood leukocyte counts. Violin plots showing the maximum, minimum and frequency of numbers of (**A**) heterophils, (**B**) monocytes, (**C**) lymphocytes and (**D**) thrombocytes in blood from laying hens vaccinated against erysipelas (light yellow) and unvaccinated laying hens (grey) in the indicated age categories. Data is shown as group plots with 86 ≥ n ≤ 100 for each group. Missing values, i.e.<100, were due to coagulation of blood samples or technical reasons at immunolabelling

**Fig. 3:**
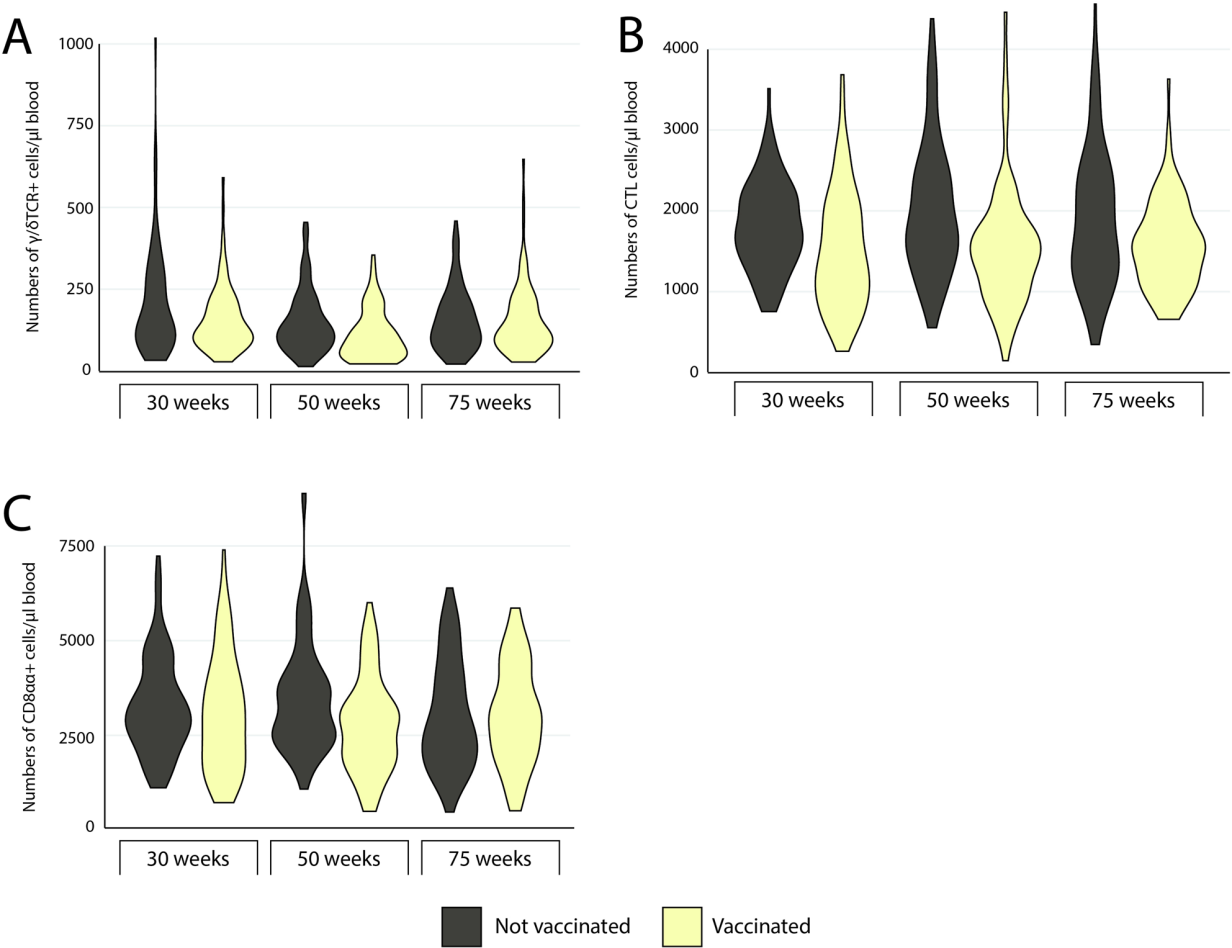
Blood lymphocyte subpopulation counts. Violin plots showing the maximum, minimum and frequency of numbers of (**A**) g/δTCR + T-cells, **B** CTL and (**C**) CD8αα+ lymphocytes in blood from laying hens vaccinated against erysipelas (light yellow) and unvaccinated laying hens (grey) in the indicated age categories. Data is shown as group plots with 82 ≥ n ≤ 100 for each group. Missing values, i.e.<100, were due to coagulation of blood samples or technical reasons at immunolabelling

**Fig. 4:**
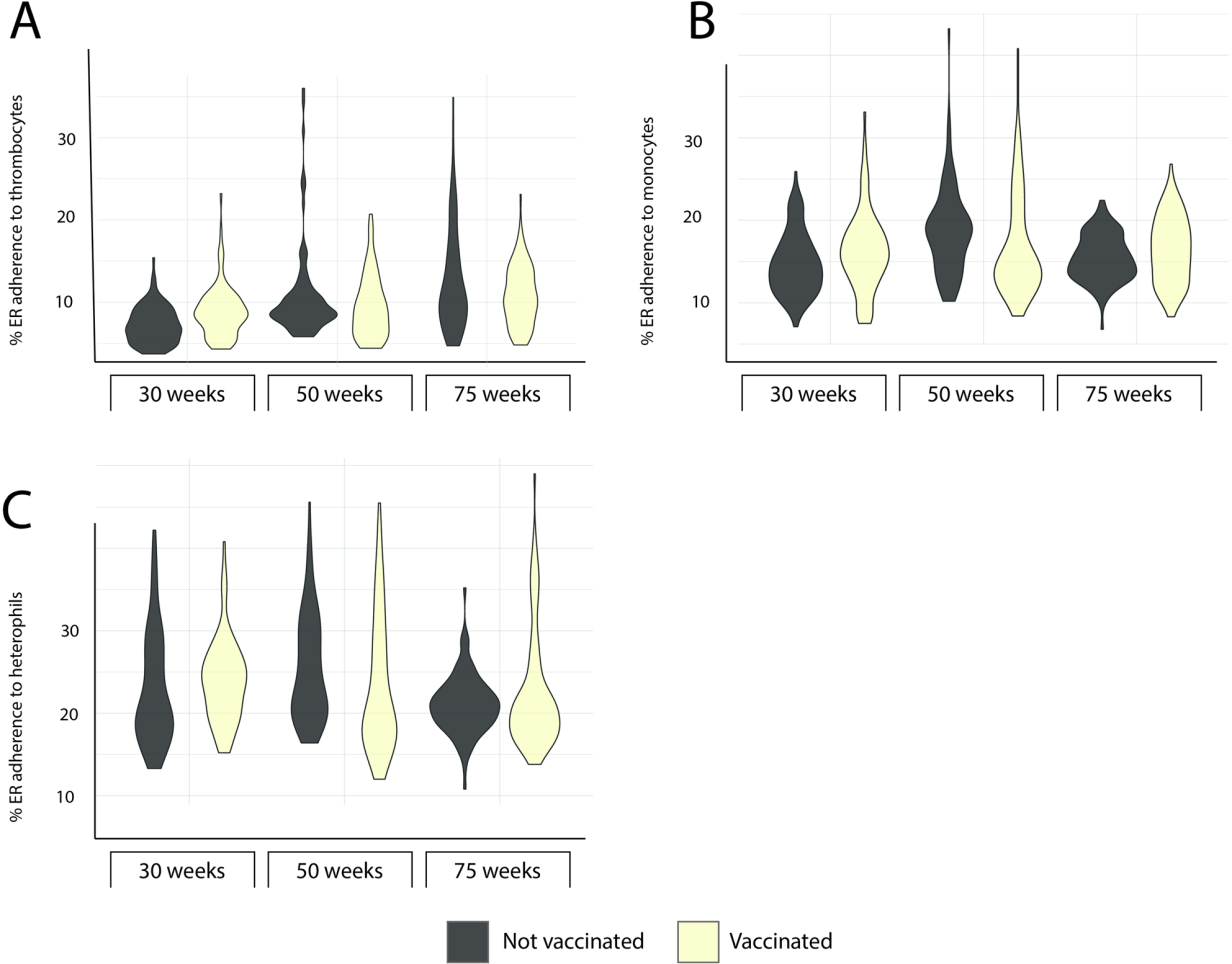
In vitro ER adherence to leukocytes. Violin plots showing the maximum, minimum and frequency of the proportion of ER adherence to (**A**) heterophils, (**B**) monocytes and (**C**) thrombocytes in whole blood cultures established from laying hens vaccinated against erysipelas (light yellow) and unvaccinated laying hens (grey) in the indicated age categories. Data is shown as group plots with 89 ≥ n ≤ 100 for each group. Missing values, i.e.<100, were due to coagulation of blood samples or technical reasons at immunolabelling

**Fig. 5:**
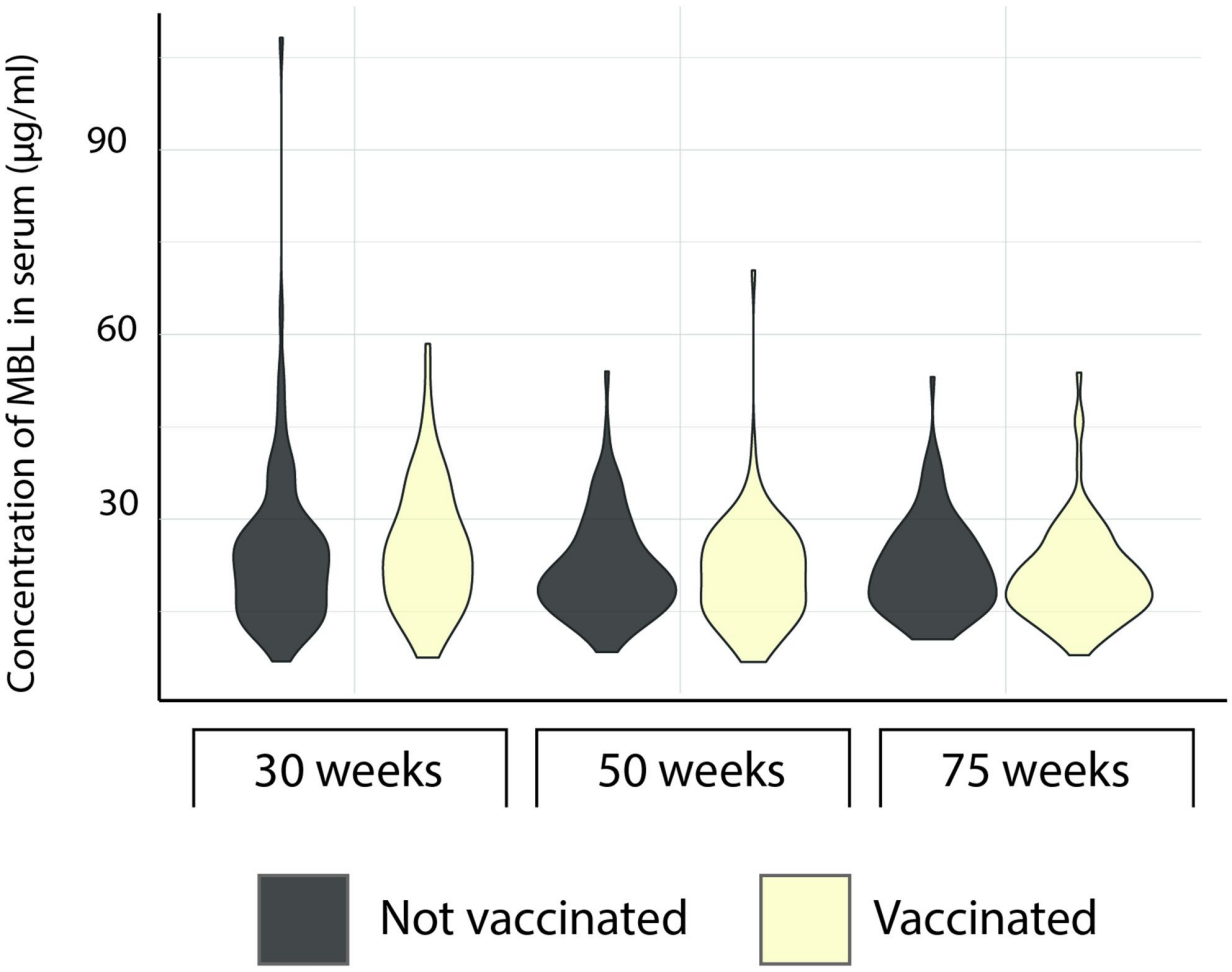
**Serum MBL** Violin plots showing the maximum, minimum and frequency of the concentration of MBL in serum from laying hens vaccinated against erysipelas (light yellow) and unvaccinated laying hens (grey) in the indicated age categories. Data is shown as group plots with 96 ≥ *n* ≤ 100 for each group. Missing values, i.e.<100, were due to insufficient amount of serum

In addition to antibodies to ER, selected immune parameters were assessed for hens in the present study, i.e., absolute numbers of leukocyte subpopulations and thrombocytes, ER adherence to leukocyte subpopulations and serum levels of MBL (Figs. 2, 3, 4 and 5).

**Fig. 6:**
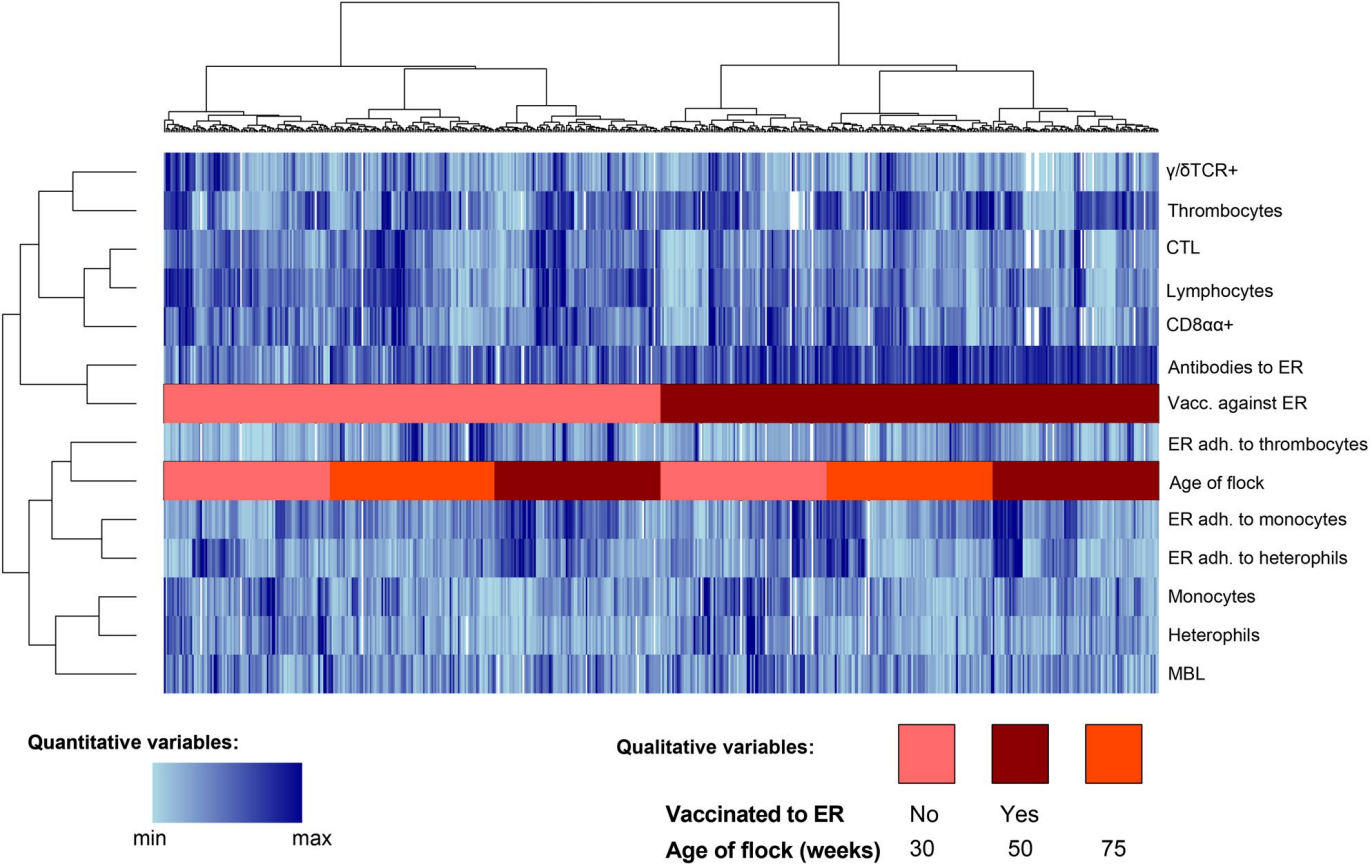
Cluster analysis. Cluster analysis including antibody levels to ER, vaccination against ER, and immune variables (numbers of γ/δTCR+ cells, thrombocytes, CTL, lymphocytes, CD8αα+ cells, monocytes and heterophils in blood, in vitro adherence of ER to thrombocytes, monocytes or heterophils, respectively, and serum concentration of MBL) across age groups from 29 Swedish layer farms. The top horizontal axis represents each sampled bird (clustered by similarity, using Gowers distance) and variables on the left vertical axis represents collected data (clustered by association)

No clear associations between the levels of IgY to ER and any of these immune parameters were identified using explorative cluster analysis (Fig. 6). However, the cluster analysis indicated two distinct clusters of variables the members of which tended to have similar patterns of high or low values in individual birds. One included vaccination and the number of lymphocytes, thrombocytes and γ/δTCR+ T-cells while the second included age category, closely clustered to ER adherence to thrombocytes, monocytes and heterophils, as well as serum MBL levels, monocyte and heterophil counts. Univariable analysis using non-parametric analysis confirmed that vaccinated hens had significantly higher absolute numbers of thrombocytes and lower absolute numbers of lymphocytes as well as the measured subpopulations of lymphocytes (γ/δTCR+ T-cells, CTL and CD8αα+ lymphocytes) compared to non-vaccinated hens (Table 3). In addition, univariable analysis indicated significant differences between the three age categories in monocyte, heterophil and γ/δTCR+ T-cell counts, which were significantly higher in younger hens compared to older hens (Table 4). However, there was large variation within age groups for all blood leukocyte parameters (Figs. 2  and 3). Especially the numbers of thrombocytes varied within age groups in a bimodal distribution (Fig. 2D).

**Table 3:** Univariable analysis comparing immune parameters for hens vaccinated against ER and hens not vaccinated against ER. Comparison made using Wilcoxon rank test. Missing observations reported for each variable (n (%), missing values were due to coagulation of blood samples, technical reasons at immunolabelling or insufficient amount of serum)

	Not vaccinated (*n* = 300)	Vaccinated (*n* = 300)	*p*-value
Heterophils/µl blood	2 954.10 [2 092.10, 4 330.50]* Missing: 6 (2.0%)	3 255.00 [2 237.60, 4 428.20] Missing: 7 (2.3%)	0.089
Lymphocytes/µl blood	11796.35 [9 565.33, 14 385.60] *Missing: 3 (1.0%)*	9666.85 [7 575.28, 11 869.43] *Missing: 6 (2.0%)*	< 0.001
Monocytes/µl blood	2122.70 [1 548.80, 2 757.70] *Missing: 4 (1.3%)*	2243.30 [1 695.72, 2 966.10] *Missing: 8 (2.7%)*	0.114
Thrombocytes/µl blood	27244.40 [14 730.92, 39 152.15] *Missing: 9 (3.0%)*	33373.65 [19 367.50, 41 119.62] *Missing: 14 (4.7%)*	0.004
g/δTCR+ cells/µl blood	139.30 [88.50, 220.95] *Missing: 3 (1.0%)*	113.85 [74.40, 191.48] *Missing: 18 (6.0%)*	0.001
CTL/µl blood	1775.00 [1 371.38, 2 337.52] *Missing: 3 (1.0%)*	1495.45 [1 056.12, 1 905.85] *Missing: 16 (5.3%)*	< 0.001
CD8αα+ cells/µl blood	2982.90 [2 275.10, 3 995.60] *Missing: 2 (0.6%)*	2949.20 [1 910.00, 3 883.00] *Missing: 15 (5%)*	0.022
ER adherence heterophils (%)	21.50 [19.00, 26.60] *Missing: 2 (0.6%)*	21.40 [18.20, 26.50] *Missing: 5 (1.7%)*	0.474
ER adherence monocytes (%)	15.60 [13.20, 18.80] *Missing: 2 (0.6%)*	15.50 [12.70, 19.15] *Missing: 5 (1.7%)*	0.981
ER adherence thrombocytes (%)	8.80 [6.80, 10.93] *Missing: 9 (3.0%)*	9.20 [7.10, 11.80] *Missing: 14 (4.5%)*	0.465
MBL µg/ml serum	21.30 [16.05, 27.10] *Missing: 4 (1.3%)*	21.30 [15.90, 27.02] *Missing: 0 (0%)*	0.711

*Values are medians and [interquartile range]

**Table 4:** Univariable analysis comparing immune parameters for hens in different age classes. Comparison made using Wilcoxon rank sum test. Missing observations reported for each variable (n (%), missing values were due to coagulation of blood samples, technical reasons at immunolabelling or insufficient amount of serum)

	30 weeks (*n* = 200)	50 weeks (*n* = 200)	75 weeks (*n* = 199)	*p*-value
Heterophils/µl blood	4 214.20 [3 115.30, 5 611.10]* Missing: 7 (3.5%)	2 600.40 [1 935.30, 3 628.60] Missing: 2 (1.0%)	2 610.70 [1 893.05, 3 850.20] Missing: 4 (2.0%)	< 0.001
Lymphocytes/µl blood	11 188.65 [8 885.48, 13 313.32] *Missing: 6 (3.0%)*	10 152.80 [7 931.65, 13 115.95] *Missing: 1 (0.5%)*	10 671.30 [8 453.50, 12 673.10] *Missing: 3 (1.0%)*	0.22
Monocytes/µl blood	2 594.35 [1 982.30, 3 519.08] *Missing: 8 (4%)*	2 155.00 [1 579.00, 2 660.25] *Missing: 1 (0.5%)*	1 874.35 [1 406.30, 2 491.22] *Missing: 3 (1.5%)*	< 0.001
Thrombocytes/µl blood	29 993.15 [13 766.70, 39 620.05] *Missing: 14 (7%)*	29 489.60 [15 064.60, 39 279.40] *Missing: 5 (2.5%)*	31 938.80 [19 192.25, 42 598.60] *Missing: 3 (1.5%)*	0.077
g/δTCR+ cells/µl blood	141.95 [93.28, 236.80] *Missing: 6 (3.0%)*	110.10 [63.80, 182.60] *Missing: 13 (6.5%)*	128.40 [83.40, 207.30] *Missing: 2 (1.0%)*	< 0.001
CTL/µl blood	1 632.20 [1 158.68, 2 119.82] *Missing: 6 (3.0%)*	1661.10 [1 309.00, 2 252.80] *Missing: 11 (5.5%)*	1 598.60 [1 211.90, 2 165.10] *Missing: 2 (1.0%)*	0.611
CD8αα+ cells/µl blood	3 029.35 [2 146.52, 4 024.95] *Missing: 4 (1.0%)*	2 955.70 [2 201.60, 3 809.40] *Missing: 11 (5.5%)*	2 805.80 [1 999.60, 4 052.50] *Missing: 2 (1.0%)*	0.648
ER adherence heterophils (%)	22.70 [18.80, 27.13] *Missing: 4 (2.0%)*	22.30 [18.70, 29.30] *Missing: 1 (0.5%)*	20.70 [18.30, 23.30] *Missing: 2 (1.0%)*	0.001
ER adherence monocytes (%)	15.05 [12.30, 18.02] *Missing: 4 (2.0%)*	16.70 [13.60, 20.75] *Missing: 1 (0.5%)*	15.60 [13.30, 18.60] *Missing: 2 (1.0%)*	0.001
ER adherence thrombocytes (%)	8.00 [5.90, 9.50] *Missing: 9 (4.5%)*	8.90 [7.30, 11.25] *Missing: 5 (2.5%)*	10.60 [8.15, 14.10] *Missing: 4 (2.0%)*	< 0.001
MBL µg/ml serum	23.10 [16.48, 29.18] *Missing: 0 (0.0%)*	20.30 [15.60, 26.42] *Missing: 4 (2.0%)*	20.40 [16.00, 25.50] *Missing: 0 (0.0%)*	0.007

*Values are medians and [interquartile range]

For ER adherence to different leukocyte populations, results showed that in general heterophils had the highest proportion of ER adherence (approx. 25%) while monocytes had a lower proportion (approx. 15%) followed by thrombocytes (approx. 10%; Fig. 4). No statistically significant effect of vaccination was observed for ER adherence to any of the leukocyte populations (Table 3). However, as suggested in the cluster analysis, there was variation between different age groups (Table 4).

For serum MBL levels, results showed that these were statistically significantly higher in hens of the youngest age category (Fig. 5; Table 4). Moreover, vaccination did not influence the MBL levels (Table 3). In addition, MBL levels and numbers of heterophils and monocytes in blood were positively correlated at the individual level (Additional file 7).

## Discussion

This study was undertaken to monitor serum antibody levels to ER in single-dose vaccinated and unvaccinated hens and to screen for potential signs of declined immune reactivity during the production period of laying hens that could explain the higher frequency of erysipelas outbreaks in older hens. The most prominent finding was that the majority of hens in the study were positive for IgY to ER regardless of vaccination status and that the titres of these antibodies were higher in older hens. This is in line with our previous observations of Swedish laying hens in which 100% of hens analysed at slaughter were positive for IgY to ER [10, 13]. In addition, a majority of hens (aged 50–90 weeks) were positive for IgY to ER also when sampled during ongoing erysipelas outbreaks [10]. In analogy with the present observations, a study of commercial chickens in New Zealand found that the proportion of birds positive for antibodies to ER increased with age [12]. These results indicate that chickens are continually exposed to ER or other ER-like bacteria in their barn and/or outdoor environment, which leads to higher antibody levels in older birds. Similar observations have been made for parrots of the endangered species kakapo (*Strigops habroptius*) where birds in their natural habitat were seropositive to ER and antibody levels to ER increased with age of the bird [39]. If it is indeed ER or if it is other apathogenic bacteria antigenically similar to ER or both that induce the high and apparently persistent ER-seropositivity of healthy laying hens observed in the current study is still under investigation. We have so far found DNA evidence that *Erysipelothrix* spp. other than ER can be found in environmental samples from indoor-housed healthy chickens positive for antibodies to ER [40]. We have also preliminary results showing that ER of the putatively less pathogenic clade 1 can be cultured from barn environmental samples from healthy laying hens with outdoor access [9].

In the present cohort we found that the levels of IgY to ER were significantly higher in hens vaccinated against erysipelas and that this difference was most prominent for the youngest age category. There was an inadvertent serotype mismatch between the ER ELISA-antigen, serotype 5, and the ER vaccine strain, serotype 2. Hence, current ER vaccine induced titres might have been relatively higher if a serotype 2 strain had been used in the ELISA. Even so, ER serotype 2 vaccines induce cross protection against ER serotype 5 challenge of mice and pigs (reviewed in [14]) and these serotypes are thus likely antigenically similar. There are very few publications on the outcome of vaccination against erysipelas in chickens. Nevertheless, one study using a commercial inactivated ER-vaccine in chickens showed a 3–4 fold increase in ER-antibody levels at the first sampling, at 21 days after the first vaccination, compared to unvaccinated chickens [41]. In that study, chickens received a second vaccination at 21 days after the first, but this did not induce any increase in antibody levels and elevated ER antibody levels compared to unvaccinated controls persisted to the end of the study at 63 days after the first vaccination. A case study in laying hens reported successful control of an erysipelas outbreak using a live attenuated vaccine but antibodies to ER were not analysed [42]. Using the same vaccine and single-dose protocol as in the present study in young, 17 or 26 days old, SPF-reared laying hen hybrids we have observed a varied outcome with respect to IgY titres to ER when assessed 2–3 weeks after vaccination with both low [25] and high (manuscript in preparation) titres compared to those induced by experimental infection of such chickens. In other bird species, reports on attenuated live or inactivated ER vaccines in turkeys show protection against ER challenge infection but antibody responses were not analysed [43–45]. For kakapo parrots an increase in ER antibody levels after vaccination with an inactivated ER-vaccine was recorded but only in birds that had low levels of ER antibodies prior to vaccination [39]. Because the vaccine ER strain used has no known distinguishing features (e.g. presence or absence of particular antigens) compared to naturally occurring ER strains it is not possible to conclusively identify vaccine induced antibody responses in the present material. It seems nonetheless likely that the higher levels of antibodies to ER in vaccinated hens compared to the unvaccinated hens observed in the present material were due to the single-dose vaccination. However, it should be noted that in Sweden vaccination against erysipelas is only performed on flocks on farms where an outbreak or outbreaks of clinical erysipelas have occurred previously. Hence, in the present study all farms where vaccination was performed had experienced outbreaks of erysipelas on some occasion prior to the present study. Consequently, it cannot be completely ruled out that the higher ER antibody levels in the vaccinated flocks were due to elevated environmental ER exposure of ER compared to farms where vaccination was not performed.

The statistical analysis showed that vaccination against ER was associated with slightly higher thrombocyte and slightly lower lymphocyte counts in the circulation. There is no evident explanation for this finding. The numerical differences in cell numbers between the two groups were rather small and the biological relevance of these minor numerical differences is questionable particularly given the high individual variation for both traits.

Adherence of live ER to phagocytic leukocytes in whole blood cultures was chosen as a simple, high throughput assay to assess the hens’ phagocytic capacity in response to ER infection. In the current setting it is primarily the collective opsonic capacity that is assessed, i.e. the sum of native opsonins and ER-specific antibodies. It should be noted that in this assay it is not possible to determine to what extent the ER bacteria were internalised or killed by the phagocytes after adherence. Antibody-mediated opsonisation for phagocytosis is a putative effector mechanism in immunity to ER infection [46]. For example, it was shown that uptake and killing of ER by both murine neutrophils and macrophages was greatly enhanced in the presence of immune serum, most likely due to ER specific antibodies [46]. Surprisingly, in the present material no overall correlation at the individual hen level was found between titres of IgY to ER and ER adherence to any of the different leukocyte populations. Nonetheless, a significant influence of flock on the levels of ER adherence was observed with e.g. up to 20% difference in ER adherent to heterophils between flocks. Previous experience with this adhesion assay indicates that antibody mediated opsonisation can indeed be detected. We have for instance seen an up to 4-fold increase in the proportion of ER adhered to heterophils when purified IgY to ER was added to the cultures (manuscript in preparation) and a 4-fold increase of *E. coli* adherence to heterophils at the time of seroconversion in *E. coli*-infected chickens [26]. Interestingly, we have also observed that antibody-mediated opsonisation in the adherence assay seems sensitive to bacterial surface antigens, e.g. serotype antigens, which is likely due to the use of intact bacteria. For ER, we observed that IgY mediated adherence to leukocytes was influenced by genetic differences between ER strains, e.g. adherence to heterophils was approximately halved when IgY raised to ER of serotype 1b was used with ER of serotype 5 compared to ER of serotype 1b or 1a (manuscript in preparation). For *E. coli*, the opsonisation effect detected in the adherence assay was serotype specific, while quantification *E. coli*-specific IgY by a sonicated whole bacteria antigen-based ELISA failed to detect these serotype differences [26]. The current ELISA to quantify IgY to ER is also based on a sonicated whole bacteria antigen and results could hence be less influenced by a mismatch between ELISA antigen strain and strains that elicited the IgY responses than corresponding mismatch in the adhesion assay. Consequently, if strain specificity of the antibodies to ER are important for leukocyte adherence and the “farm ER flora” differed between flocks, varying antigenic compatibility to the ER strain used in the assay could explain some of the flock variation for this trait. For ER outbreak strains, we have observed that isolates from different farms show a large genetic variation while multiple isolates from the same flock and from different flocks on the same farm often were closely related when outbreaks clustered in time [2]. This observation could thus support that a variation in “farm ER flora” could exist also between healthy flocks. Taken together, it cannot be excluded that antibodies to ER were involved in bacterial adherence to leukocytes and causing some of the variation observed for this trait.

Blood leukocyte counts were monitored in the present study as an indirect measure of health and immunological status. The most prominent of these results was the significant influence of age on the numbers of circulating heterophils and monocytes with hens in the youngest age category having the highest numbers. Increased heterophil and monocyte numbers are associated with infectious and inflammatory diseases, e.g. bacterial infections [17], and/or a general high pathogen load. Moreover, heterophil numbers may also increase in the circulation during stress (reviewed in [18]) while monocyte numbers have both been shown to increase [47] and decrease during stress in chickens [48]. Hence, our results could reflect both a higher presence of infections and/or higher levels of stress in the younger hens. However, we also monitored serum MBL levels in this cohort. This acute phase protein is synthesised in response to infections and increased serum MBL levels have been monitored after e.g. viral [21, 23, 49] and bacterial infections of chickens [22, 25]. In the present study, serum MBL concentration was significantly influenced by age with the highest amount in the youngest age group and a positive correlation at the individual hen level between MBL amount and numbers of heterophils and monocytes was shown. This indicates that the higher heterophil and monocyte numbers in the youngest age category were more likely due to a higher pathogen load than to stress. Moreover, the second cluster identified by the cluster analysis that associated age category with ER adherence to thrombocytes, monocytes and heterophils, as well as serum MBL levels, monocyte and heterophil counts was likely also a reflection of this.

Among lymphocyte subpopulations, γ/δTCR+ T-cells have been pointed out as important in the immunity to bacterial infections (reviewed in [17]). For example, a reduction of the γ/δTCR+ T-cell population in the reproductive tract at point-of-lay has been implicated in the increased susceptibility of hens to *Salmonella* infections at this production stage [50]. We were therefore interested to see if changes in this cell population could indicate why older flocks seem more susceptible to erysipelas outbreaks. Indeed, the number of γ/δTCR+ T-cells were significantly higher in the youngest age category. The numerical differences between age categories in γ/δTCR+ T-cell numbers were however low. This observation may thus be a reflection of ageing but could likewise, and probably more likely, be a reflection of subclinical infections as discussed above. On the whole, even though flocks in the oldest age category showed signs of ageing such as lower laying rate and decreased water consumption, hens in this category did not stand out as immune compromised in any of the traits assessed in the present study.

To conclude, results from this study showed that antibodies to ER were prevalent in all hens regardless of vaccination status or age category. The ER antibody levels were higher in vaccinated compared to unvaccinated hens and higher in older both vaccinated and unvaccinated hens. Moreover, we found that hens in the youngest age category likely experienced a higher general pathogen load/level of subclinical infections as indicated by altered leukogram patterns and increased MBL-levels. Moreover, we found no evidence to suggest that antibody responses to single-dose vaccination should fail or why older flocks seem more susceptible to erysipelas outbreaks. Our results strongly suggest that more insight into the source of ER exposure in the chicken environment is needed. Given the high prevalence of antibodies recognising ER among laying hens that are likely vulnerable to erysipelas outbreaks, more insight into the role of antibodies in the chicken immune response to ER infection is also clearly required. 

## Supplementary Information

Additional file 1. Description of categorical and numerical variables in the study

Additional file 2. Monoclonal antibodies used for immunolabelling and combinations (panels) used for whole blood leukocyte counts and whole blood cultures

Additional file 3. Gating strategy for enumeration of leukocytes with panel 1 in whole blood samples by identification of counting beads, heterophils, monocytes, thrombocytes, and lymphocytes through singlet gating, FSC/SSC characteristics and using CD45-PerCpCy5.5, CD41/61-Fitc, and MRC1L-B-RPE. From all events in initial dot-plot in A) counting beads were identified as high fluorescent in B). From all events in A) gating through FSC-H vs FSC-A was performed in C) to identify singlets. From this gate high CD45 expressing events (leukocytes) and low SCC-A and medium to high CD45 expressing events (potential thrombocytes) were gated in D). From the CD45 gate in D) events were defined according to FSC and SSC characteristics in E) and high SSC events were defined in P1. P1 events were defined according to CD45 expression in F) and high CD45 expressing events were defined in P2. P2 events were defined according to FSC and SSC characteristics as heterophils in G). From the CD45 gate in D) events were defined according to CD41/61 and MRC1L-B expression in H) and high CD41/61 events were defined as thrombocytes. MCR1L-B positive events from H) were defined according to FSC and SSC characteristics as monocytes in I). Non-thrombocyte events defined in H) were defined according to FSC and SSC characteristics as lymphocytes in J). A representative blood sample from an unvaccinated 50-week-old hen is shown. The antibody panel is described in Additional file 2

Additional file 4. Gating strategy for enumeration of leukocytes with panel 2 in whole blood samples by identification of counting beads, TCRγ/ẟ+, TCRγ/ẟ-CD8αβ+ (CTL), TCRγ/ẟ-CD8αα+, cells through singlet gating, FSC/SSC characteristics and using CD41/61-Fitc, TCRγ/ẟ-PerCp/Cy5.5, CD8α-APC, and CD8β-RPE. From all events in initial dot-plot in A) counting beads were identified as high fluorescent in B). From all events gating through FSC-H vs FSC-A was performed in C) to identify singlets. Thrombocytes were excluded from singlets based on CD41/61 expression in D) In E) Non-thrombocytes were defined as “lymphocytes” according to FSC and SSC characteristics. “Lymphocytes” were defined according to TCRγ/ẟ expression as TCRγ/ẟ+ in F) Non-TCRγ/ẟ+ events in F) were defined according to CD8β expression in G) and TCRγ/ẟ-CD8αβ+ (CTL) and TCRγ/ẟ-CD8αα+ cells were identified. A representative blood sample from an unvaccinated 50-week-old hen is shown. The antibody panel is described in Additional file 2

Additional file 5. Gating strategy for identification of leukocytes and ER adherence to leukocytes with panel 1 in whole blood cultures by identification heterophils, monocytes, and thrombocytes, through singlet gating, FSC/SSC characteristics and using CD45-PerCpCy5.5, CD41/61-Fitc, MRC1L-B-RPE and ER labelled with Far Red (ER-FarRed). From all events in initial dot-plot in A) gating through FSC-H vs FSC-A was performed in B) to identify singlets. From this gate high CD45 expressing events (leukocytes) and low SCC-A and medium to high CD45 expressing events (potential thrombocytes) were gated in C). From the CD45 gate in C) events were defined according to FSC and SSC characteristics in D) and high SSC events were defined in P1. P1 events were defined according to CD45 expression in E) and high CD45 expressing events were defined in P2. P2 events were defined according to FSC and SSC characteristics as heterophils in F). Heterophils defined in F) were assessed for ER adhesion in G). Heterophils from the same hen in a control culture incubated without ER-FarRed are shown in L. From the CD45 gate in C) events were defined according to CD41/61 and MRC1L-B expression in H) and high CD41/61 events were defined as thrombocytes. MCR1L-B positive events from H) were defined according to FSC and SSC characteristics as monocytes in I). Monocytes defined in I) were assessed for ER adhesion in J). Monocytes from the same hen in a control culture incubated without ER-FarRed are shown in M. Thrombocytes defined in H) were assessed for ER adhesion in K). Thrombocytes from the same hen in a control culture incubated without ER-FarRed are shown in N

Additional file 6. Titres to ER in serum from laying hens vaccinated against erysipelas (white boxes) and unvaccinated laying hens (grey boxes) in the indicated flocks and age categories. Data is shown as flock box plots with 17≥n≤20 for each flock. Missing values, i.e.<20, were due to insufficient amount of serum. Boxes enclose 50% of the data with the median value displayed as a horizontal line, the limits of the box represent the upper and lower quartile. Whiskers mark the maximum and minimum values, excluding outliers. Open circles represent outliers defined as values greater than the upper quartile, or smaller than the lower quartile, + 1.5x the interquartile distance

Additional file 7. Correlation (Pearson coefficient) of immunological parameters on individual hen level

## Data Availability

All data generated and analysed in this study are included in the article text, tables, figures and additional files. The raw datasets used and/or analysed during the current study are available from the corresponding author on reasonable request.
