# Supplementary material for: Immune parameters monitored during the production period of laying hens managed with or without single-dose vaccination against erysipelas
Source: BMC Vet Res. 2026 May 1;22:263. doi: 10.1186/s12917-026-05512-w (PMC13137716; doi:10.1186/s12917-026-05512-w)
Supplement: Supplementary file 1 — Additional file 1. Description of categorical and numerical variables in the study [file 12917_2026_5512_MOESM1_ESM.doc]

**Additional file 1.** Description of categorical and numerical variables in the study

| Categorical | Numerical |
| --- | --- |
| Flock | Age^a^ (weeks)** |
| Age group | Facility in use^b^ (years) |
| Vaccination status | Outdoor time^c^ (weeks) |
| Production (organic/free-range) | Cumulative mortality rate^d^ (%)*** |
| Breeder* | Laying rate^e^ (%) |
| Acidification of water (yes/no) | Feed consumption^e^ (g/chicken/day)*** |
| Vitamin supplements (yes/no) | Water consumption^e^ (ml/chicken/day)*** |
| De-worming against *Ascaridia galli* (yes/no) | Body weight^e^ (g)**** |
| Treatment against *Dermanyssus gallinae* (yes/no) | Age at vaccination against other pathogens (weeks)* |
| Vaccination against other pathogens than ER (yes/no) |  |
| Route of ER vaccination (intra-muscular/subcutaneous)* |  |
| Vaccinator for administering ER vaccine* |  |

* Excluded due to positivity violation / lack of overlap at the cluster level (leading to non-identifiability)
** Was treated as a categorical variable due to the study design with three age groups (Age group)

***Excluded due to high number of missing values after sensitivity analysis

**** Excluded due to high number of missing values

a – Age of flock at sampling

b – Number of years the chicken house had been in use at the time of sampling

c – Number of weeks the flock had had access to the outdoor range (due to weather conditions) during the 2019 season at the time of sampling

d – Total mortality recorded for the flock from placement until the time of sampling

e - Laying rate, feed and water consumption and hen body weight recorded at the time of sampling
