# Supplementary material for: Immune parameters monitored during the production period of laying hens managed with or without single-dose vaccination against erysipelas
Source: BMC Vet Res. 2026 May 1;22:263. doi: 10.1186/s12917-026-05512-w (PMC13137716; doi:10.1186/s12917-026-05512-w)
Supplement: Supplementary file 2 — Additional file 2. Monoclonal antibodies used for immunolabelling and combinations (panels) used for whole blood leukocyte counts and whole blood cultures [file 12917_2026_5512_MOESM2_ESM.doc]

**Additional file 2**. Monoclonal antibodies used for immunolabelling and combinations (panels) used for whole blood leukocyte counts and whole blood cultures

| Abbreviation | Clone | Specificity | Fluorochrome | Panel 1 | Panel 2 |
| --- | --- | --- | --- | --- | --- |
| MRC1L-B-RPE | KUL01^a^ | Chicken mannose receptor MRC1L-B [1] | R-phycoerythrin^c^ | X | - |
| CD41/61-Fitc | 11C3^b^ | Chicken CD41/61 intergrin (GPIIb-IIIa) | Fluorescein^c^ | X | X |
| CD45-PerCp/Cy5.5 | UM16-6^b^ | Chicken CD45, all isoforms [2] | Peridinin chlorophyll-cyanine 5.5^d^ | X | - |
| TCRγ/δ-PerCp/Cy5.5 | TCR-1^a^ | Chicken γ/δ T-cell receptor | Peridinin chlorophyll-cyanine 5.5^d^ | - | X |
| CD8β-RPE | EP42^a^ | β-chain of chicken CD8 | R-phycoerythrin^c^ | - | X |
| CD8α-APC | CT-8^a^ | α-chain of chicken CD8 | Allophycocyanin^c^ | - | X |

X: used in panel, -:not used in panel

a Purchased from Southern Biotech.

b Purchased from Bio-Rad Antibodies.

c Fluorochrome conjugated by manufacturer.

d Fluorochrome conjugated using Lightning-Link® conjugation kits (Abcam) according to the manufacturer’s protocol.

**References**

1. Staines K, Hunt LG, Young JR, Butter C (2014) Evolution of an expanded mannose receptor gene family. PLoS One 9 (11):e110330 doi:10.1371/journal.pone.0110330

2. Huhle D, Hirmer S, Göbel TW (2017) Splenic γδ T cell subsets can be separated by a novel mab specific for two CD45 isoforms. Dev Comp Immunol 77:229-240 doi:10.1016/j.dci.2017.08.013
