# Supplementary figures and images for: Immune parameters monitored during the production period of laying hens managed with or without single-dose vaccination against erysipelas
Source: BMC Vet Res. 2026 May 1;22:263. doi: 10.1186/s12917-026-05512-w (PMC13137716; doi:10.1186/s12917-026-05512-w)

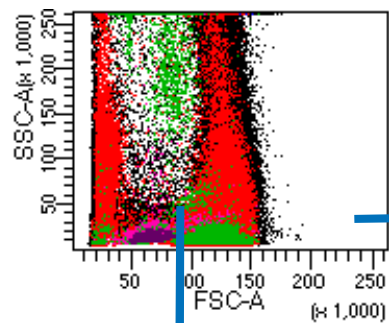

**A**

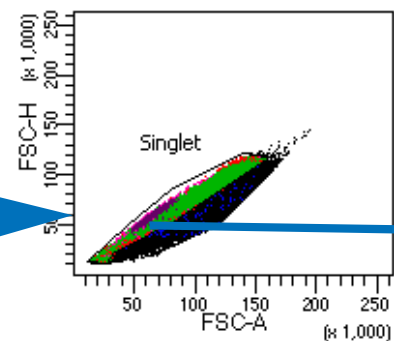

**C**

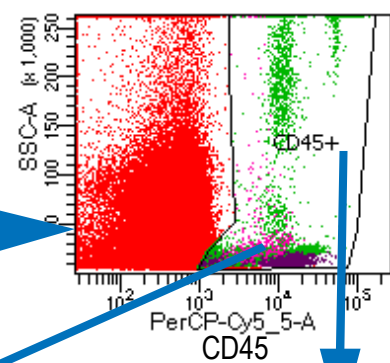

**D**

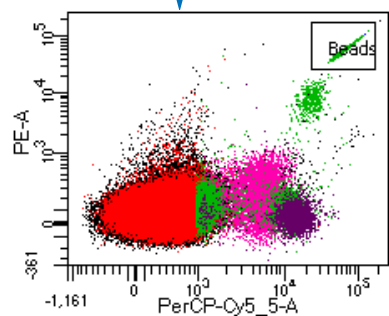

**B**

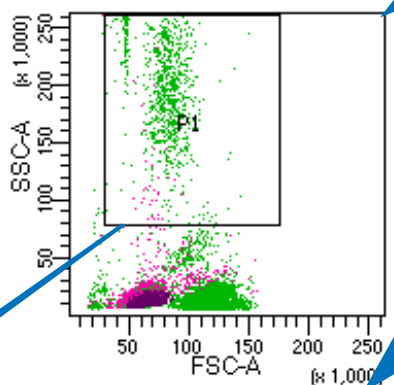

**E**

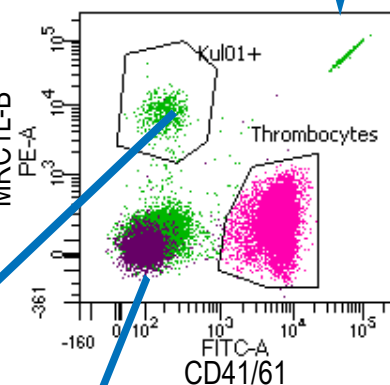

**H**

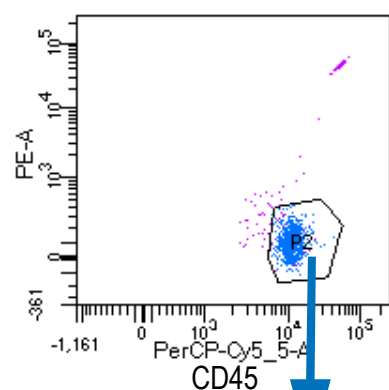

**F**

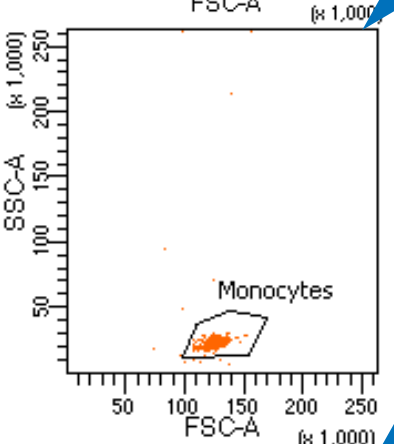

**I**

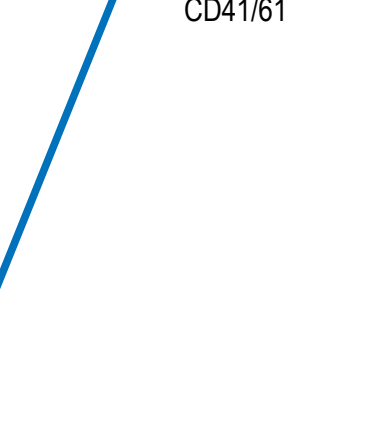

**J**

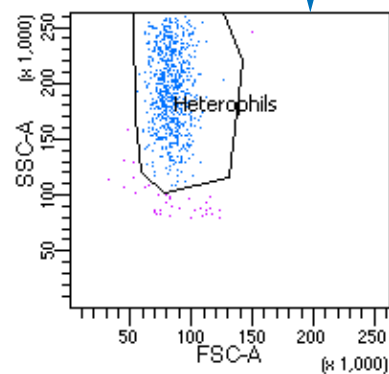

**G**

Supplement: Supplementary file 3 — Additional file 3. Gating strategy for enumeration of leukocytes with panel 1 in whole blood samples by identification of counting beads, heterophils, monocytes, thrombocytes, and lymphocytes through singlet gating, FSC/SSC characteristics and using CD45-PerCpCy5.5, CD41/61-Fitc, and MRC1L-B-RPE. From all events in initial dot-plot in A) counting beads were identified as high fluorescent in B). From all events in A) gating through FSC-H vs FSC-A was performed in C) to identify singlets. From this gate high CD45 expressing events (leukocytes) and low SCC-A and medium to high CD45 expressing events (potential thrombocytes) were gated in D). From the CD45 gate in D) events were defined according to FSC and SSC characteristics in E) and high SSC events were defined in P1. P1 events were defined according to CD45 expression in F) and high CD45 expressing events were defined in P2. P2 events were defined according to FSC and SSC characteristics as heterophils in G). From the CD45 gate in D) events were defined according to CD41/61 and MRC1L-B expression in H) and high CD41/61 events were defined as thrombocytes. MCR1L-B positive events from H) were defined according to FSC and SSC characteristics as monocytes in I). Non-thrombocyte events defined in H) were defined according to FSC and SSC characteristics as lymphocytes in J). A representative blood sample from an unvaccinated 50-week-old hen is shown. The antibody panel is described in Additional file 2 [file 12917_2026_5512_MOESM3_ESM.pdf]

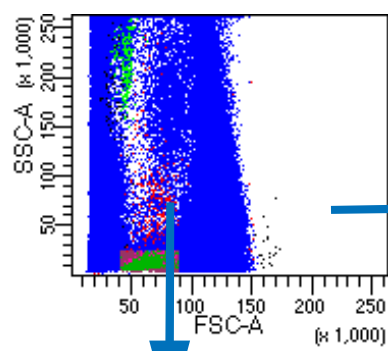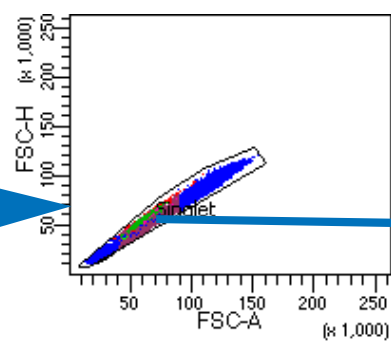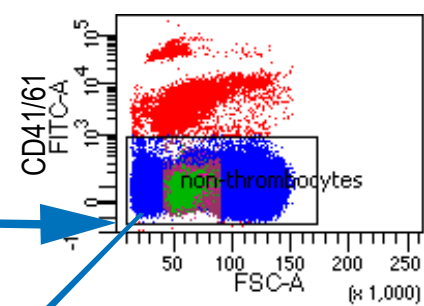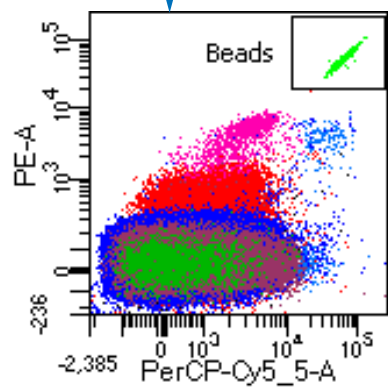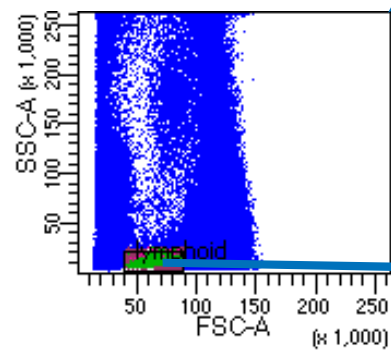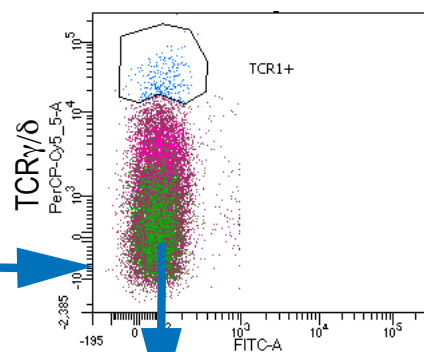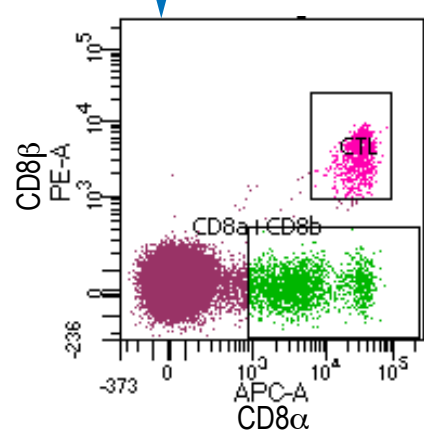

Supplement: Supplementary file 4 — Additional file 4. Gating strategy for enumeration of leukocytes with panel 2 in whole blood samples by identification of counting beads, TCRγ/ẟ+, TCRγ/ẟ-CD8αβ+ (CTL), TCRγ/ẟ-CD8αα+, cells through singlet gating, FSC/SSC characteristics and using CD41/61-Fitc, TCRγ/ẟ-PerCp/Cy5.5, CD8α-APC, and CD8β-RPE. From all events in initial dot-plot in A) counting beads were identified as high fluorescent in B). From all events gating through FSC-H vs FSC-A was performed in C) to identify singlets. Thrombocytes were excluded from singlets based on CD41/61 expression in D) In E) Non-thrombocytes were defined as “lymphocytes” according to FSC and SSC characteristics. “Lymphocytes” were defined according to TCRγ/ẟ expression as TCRγ/ẟ+ in F) Non-TCRγ/ẟ+ events in F) were defined according to CD8β expression in G) and TCRγ/ẟ-CD8αβ+ (CTL) and TCRγ/ẟ-CD8αα+ cells were identified. A representative blood sample from an unvaccinated 50-week-old hen is shown. The antibody panel is described in Additional file 2 [file 12917_2026_5512_MOESM4_ESM.pdf]

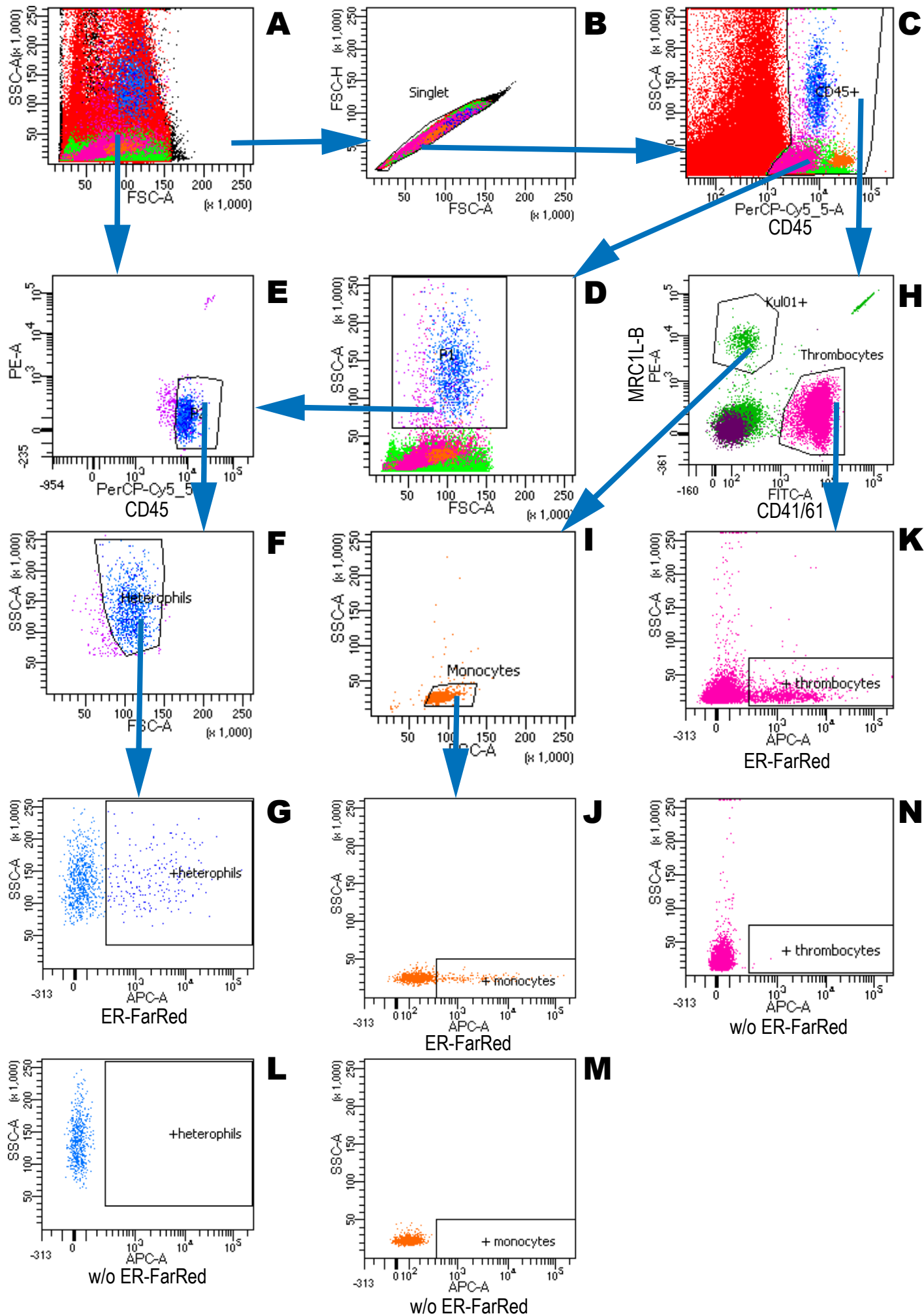

Supplement: Supplementary file 5 — Additional file 5. Gating strategy for identification of leukocytes and ER adherence to leukocytes with panel 1 in whole blood cultures by identification heterophils, monocytes, and thrombocytes, through singlet gating, FSC/SSC characteristics and using CD45-PerCpCy5.5, CD41/61-Fitc, MRC1L-B-RPE and ER labelled with Far Red (ER-FarRed). From all events in initial dot-plot in A) gating through FSC-H vs FSC-A was performed in B) to identify singlets. From this gate high CD45 expressing events (leukocytes) and low SCC-A and medium to high CD45 expressing events (potential thrombocytes) were gated in C). From the CD45 gate in C) events were defined according to FSC and SSC characteristics in D) and high SSC events were defined in P1. P1 events were defined according to CD45 expression in E) and high CD45 expressing events were defined in P2. P2 events were defined according to FSC and SSC characteristics as heterophils in F). Heterophils defined in F) were assessed for ER adhesion in G). Heterophils from the same hen in a control culture incubated without ER-FarRed are shown in L. From the CD45 gate in C) events were defined according to CD41/61 and MRC1L-B expression in H) and high CD41/61 events were defined as thrombocytes. MCR1L-B positive events from H) were defined according to FSC and SSC characteristics as monocytes in I). Monocytes defined in I) were assessed for ER adhesion in J). Monocytes from the same hen in a control culture incubated without ER-FarRed are shown in M. Thrombocytes defined in H) were assessed for ER adhesion in K). Thrombocytes from the same hen in a control culture incubated without ER-FarRed are shown in N [file 12917_2026_5512_MOESM5_ESM.pdf]

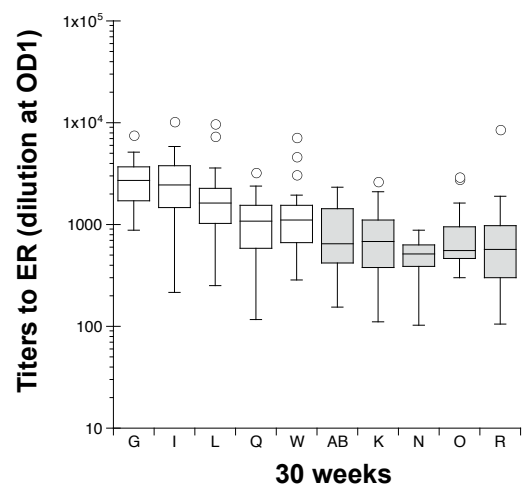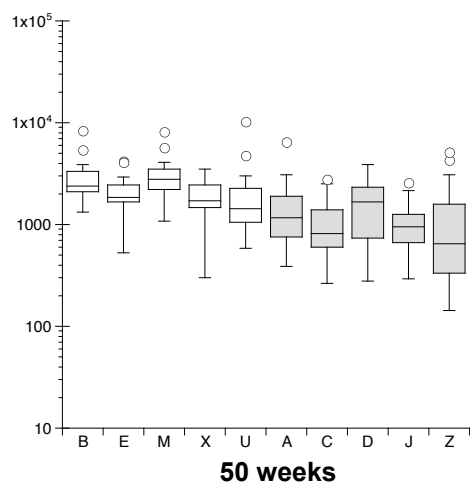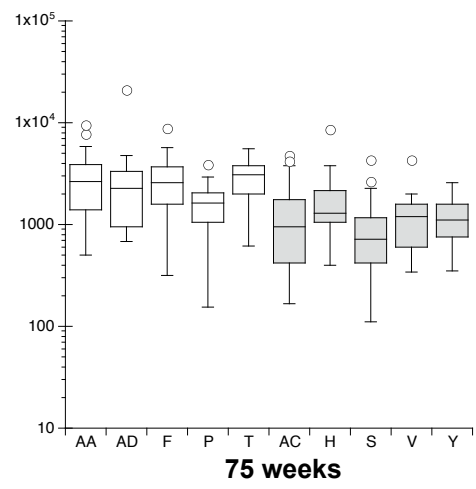

Supplement: Supplementary file 6 — Additional file 6. Titres to ER in serum from laying hens vaccinated against erysipelas (white boxes) and unvaccinated laying hens (grey boxes) in the indicated flocks and age categories. Data is shown as flock box plots with 17≥n≤20 for each flock. Missing values, i.e.<20, were due to insufficient amount of serum. Boxes enclose 50% of the data with the median value displayed as a horizontal line, the limits of the box represent the upper and lower quartile. Whiskers mark the maximum and minimum values, excluding outliers. Open circles represent outliers defined as values greater than the upper quartile, or smaller than the lower quartile, + 1.5x the interquartile distance [file 12917_2026_5512_MOESM6_ESM.pdf]

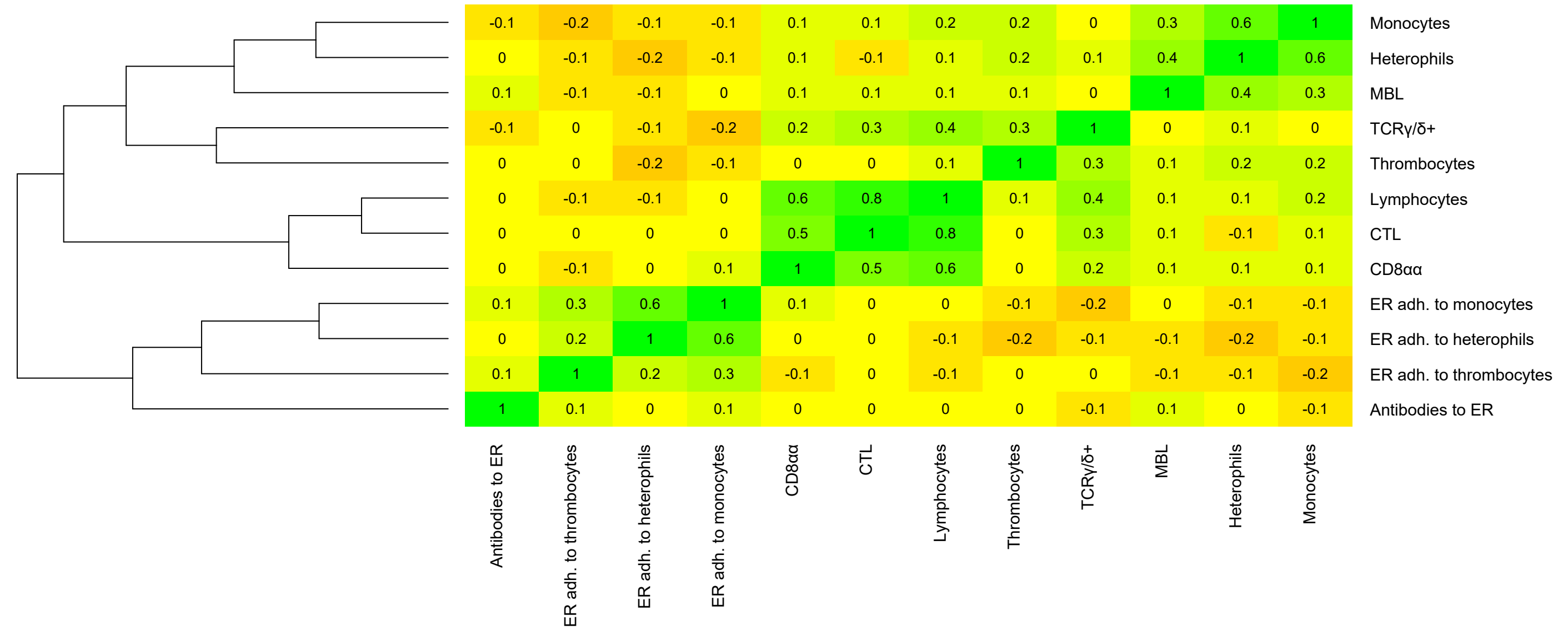

Supplement: Supplementary file 7 — Additional file 7. Correlation (Pearson coefficient) of immunological parameters on individual hen level [file 12917_2026_5512_MOESM7_ESM.pdf]
